# Rumo zhentong Yin for blood-stasis-type osteoporotic pain: protocol of a randomized, single-blind, controlled trial

**DOI:** 10.3389/fragi.2026.1876169

**Published:** 2026-09-11

**Authors:** Qian Zhang, Yuanjie Song, Bailu Niu, Mianjie Liang, Jingjing Yang, Yanjie Tong, Hui Wang

**Affiliations:** 1 Department of Geriatrics, Xi’an Hospital of Traditional Chinese Medicine, Xi’an, China; 2 School of Traditional Chinese Medicine, Shaanxi University of Chinese Medicine, Xi’an, Shaanxi, China

**Keywords:** blood-stasis syndrome, osteoporotic pain, randomized controlled trial, Rumo zhentong Yin, traditional Chinese medicine

## Abstract

**Background:**

Osteoporosis (OP) is a metabolic skeletal disease characterized by reduced bone mass and micro-architectural deterioration, with Osteoporotic pain (OPP) as its most common clinical symptom, which markedly impairs quality of life. Modern pharmacologic treatments’ limitations are obvious with population aging, so the cost-effective interventions for OPP are urgently needed. As an empirical formula, Rumo zhentong Yin has been created by the national renowned veteran Traditional Chinese Medicine (TCM) practitioner Yao Shujin for blood-stasis pain. Hence, this trial was designed to investigate the efficacy of Rumo zhentong Yin in treating blood-stasis-type OPP.

**Methods:**

A randomized, single-blind, controlled trial was designed to evaluate the efficacy of Rumo zhentong Yin combined with standard therapy for OPP. Eligible OPP patients were recruited and randomly assigned (1:1) to form either the control or treatment group. Both groups received standard treatment. The intervention lasted 3 months, with follow-up evaluations every 4 weeks. Primary outcome was pain scores; secondary outcomes included bone mineral density (BMD), bone-turnover markers, inflammatory cytokines, blood-stasis syndrome score and hematological indices.

**Anticipated Results:**

To our knowledge, this trial is the first clinical study of Rumo zhentong Yin for OPP, with the primary objective to provide robust clinical evidence supporting the integration of Traditional Chinese and Western medicine for OPP.

**Trial Registration:**

The trial was registered at the International Traditional Medicine Clinical Trial Registry (www.itmctr.ccebtcm.org.cn) on 2026-01-08 (ITMCTR2026000740).

## Introduction

As global aging accelerates, osteoporosis (OP) has become one of the emblematic disorders of the aging era. Characterized by continuous loss of bone mass and deterioration of bone micro-architecture, OP leads to increased skeletal fragility and a higher risk of fracture. Clinically, it presents as low-back pain, aching in the extremities, loss of height and, in many cases, fragility fractures (Chinese Medical Association Branch of Osteoporosis and Bone Mineral Diseases, 2022). More than 200 million people are affected globally, with women bearing the greatest burden (National Center for Chronic Noncommunicable Diseases, 2021).

Osteoporotic pain (OPP) is the chief complaint that brings patients to medical attention and the principal factor limiting their daily activities. About 85% of individuals with OP report pain (Dinges, 2026), comprising both acute fracture-related nociceptive pain and chronic persistent pain (Vellucci et al., 2018). This substantially compromises quality of life in elderly patients who frequently have multiple comorbidities and may precipitate anxiety, depression and other psychological problems (Lisowska et al., 2018).

Current pharmacologic management includes anti-osteoporotic agents and non-steroidal anti-inflammatory drugs (NSAIDs). These therapies, however, carry risks such as hypersensitivity reactions, fever, peptic ulceration and increased cardiovascular-thrombotic events. Traditional Chinese medicine (TCM) offers a safer and effective alternative. Herbal formulae not only relieve pain but also correct underlying constitutional imbalances, reduce dependence on analgesics, minimize adverse reactions and improve overall quality of life (Shima et al., 2016).

Blood stasis is not only a causative factor of osteoporotic pain (OPP) but also a pathological consequence of the condition itself. As a result, blood-stasis syndrome (BSS) represents one of the most commonly observed traditional Chinese medicine (TCM) patterns in patients with osteoporotic pain (OPP). Its cardinal manifestations include aching, weakness and stabbing pain localized in the lower back and knees—or throughout the body—together with limitation of movement. Applying the Practical Diagnostic Criteria for Blood-Stasis Syndrome (Table 1) markedly enhances the accuracy and reliability of BSS identification in clinical practice.

**TABLE 1:** Schedule of trial.

Study phase Tests		Screening period (Day)	Treatment period (Day)		
Visit window		−3∼0	Day 30 of treatment	Day 60 of treatment	Day 90 of treatment
Basic history taking					
Informed-consent signature		×	​	​	​
Demographics		×	​	​	​
General data form		×	​	​	​
Medical and prior-treatment history		×	​	​	​
Inclusion/exclusion check		×	​	​	​
Randomisation and enrolment		×	​	​	​
Screening variables					
Bone-mineral density (BMD)		×	​	​	​
Treatment administration					
Test group		​	×	×	×
Control group		​	×	×	×
Efficacy end-points (assessment visits)					
BMD measurement		×	​	​	×
TCM syndrome score		×	×	×	×
Pain score (VAS)		×	×	×	×
Bone-turnover markers (β-CTX, PINP)		×	×	×	×
Inflammatory cytokines (TNF-α, IL-1)		×	×	×	×
Blood-stasis syndrome score		×	×	×	×
Blood-cell indices		×	×	×	×
Safety observations					
Height		×	​	​	×
Blood pressure		×	​	​	×
Electrocardiogram		×	​	​	×
Haematology		×	​	​	×
Liver function		×	×	×	×
Renal function		×	×	×	×
Coagulation profile		×	​	​	×
Adverse-event log		​	×	×	×
Other items					
Concomitant medication / therapy		×			
Study closeout	Withdrawals and drop-outs	×			
	Compliance and safety evaluation form	×			
	Abnormalfindings record	×			
	Studycompletion status	×			
Review & sign-off		×			

Rumo zhentong Yin—composed of Ruxiang (frankincense), Moyao (myrrh), Xuejie (dragon’s blood), Jiangxiang (dalbergia wood), Sumu (sappan wood), Gansong (nardostachys), Xixin (asarum), and Tubiechong (ground beetle)—is an empirical formula created by national TCM master Yao Shujin for blood-stasis pain. Although preclinical studies support the anti-inflammatory, analgesic, and bone-protective effects of its ingredients, robust clinical evidence is still lacking (Shima et al., 2016; Lisowska et al., 2018).

## Methods and analysis

### Study design and setting

**FIGURE 1:**
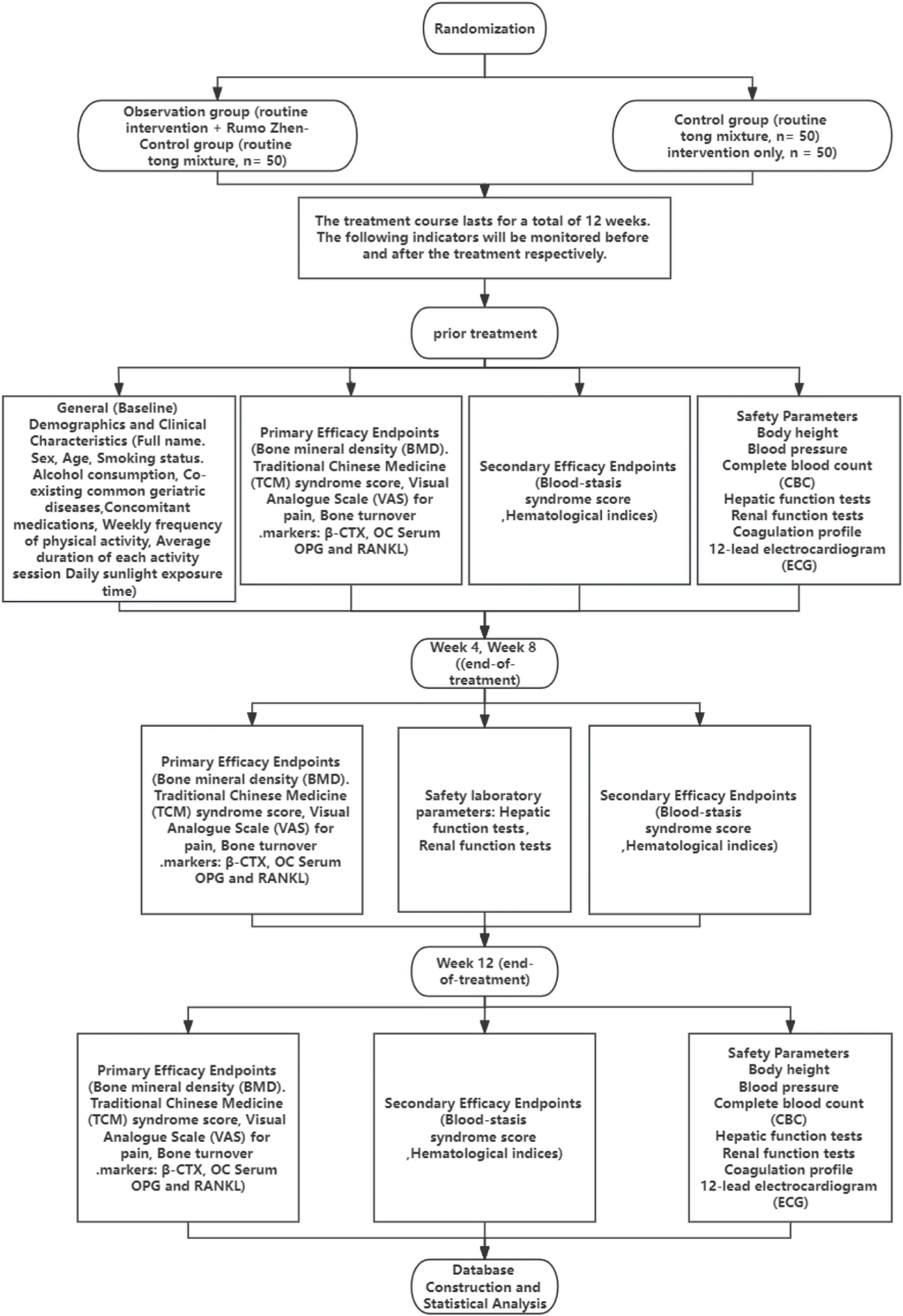
Trial flow chart.

The protocol was approved by the Medical Ethics Committee of Xi’an Hospital Affiliated to Shaanxi University of Chinese Medicine (approval No. LLSCYJ2025085) and registered at the International Traditional Medicine Clinical Trial Registry (ITMCTR2026000740). The experimental flowchart is shown in Figure 1.

This is a single-centre, two-arm, single-blind, randomized controlled trial. A total of 100 participants will be recruited through advertisements and referrals by outpatient and inpatient clinicians. Eligible patients will be informed about the study when they attend the outpatient clinic or are admitted to the hospital. A detailed face-to-face interview will be conducted to explain the study’s purpose, interventions, assessment procedures, and follow-up schedule. Following the provision of written informed consent, participants will be randomly assigned (1:1) to either the treatment group or control group. Both groups will receive standard care throughout the intervention period. In addition, the treatment group will receive Rumo zhentong Yin decoction, while the control group will receive standard care alone. The intervention will continue for up to 3 months, unless a participant withdraws consent, is lost to follow-up, or dies. Follow-up visits will be scheduled every 4 weeks, as outlined in Table 1.

### Participants

Recruitment will be conducted through outpatient and inpatient departments of geriatrics, spine, and trauma at Xi’an Hospital of TCM.

### Inclusion criteria

Participants are eligible if they meet ALL of the following: Voluntary agreement to take part and signed informed consent.

Diagnosis of primary osteoporosis according to Western-medicine criteria. TCM syndrome differentiation of “blood-stasis syndrome”. ndividuals aged between 45 and 70 years, regardless of gender.Low-back or limb-joint pain for ≥3 months.

### Exclusion criteria

Participants will be excluded if ANY of the following apply:

Fracture within the past 6 months.

Concomitant endocrine or metabolic diseases (hyperparathyroidism, Cushing’s syndrome, hyperthyroidism, gonadal hyper-function, diabetes), hematological diseases (myeloma, leukaemia), severe gastrointestinal diseases, or psychiatric/neurological disorders. Low-back pain attributable to lumbar-disc herniation, cervical spondylosis, or other clear causes.

Severe cardio-cerebrovascular disease, hepatic or renal insufficiency. Psychosis or inability to give informed consent. Participation in another clinical trial or completion of another drug trial within 3 months.

### Withdrawal/discontinuation criteria

Participants will be withdrawn or discontinued if ANY of the following occur: violation of inclusion criteria or fulfillment of any exclusion criterion during the study. Receipt of study treatment for fewer than 30 consecutive days or evidence of poor compliance. Occurrence of serious side-effects, adverse reactions, or allergic reactions. Initiation or modification of concomitant therapies that could influence outcomes without prior approval from the investigator. Withdrawal of informed consent by the participant or their legally authorized representative. Significant disease progression or the development of a new medical condition requiring urgent or alternative.

### Drop-out criteria

Participants are considered drop-outs if ANY of the following occur: Failure to attend scheduled visits on time.

Are lost to follow-up.

Voluntarily withdraw before completing the full course.

Do not adhere to the protocol to an extent that efficacy evaluation is compromised.

### Intervention

Basic therapy: Alendronate sodium (MSD PHARMA [SINGAPORE] PTE.LTD.; Import Drug Registration No. H20160100; 70 mg) 70 mg orally once weekly; Calcium carbonate D_3_ (Wyeth Pharmaceutical Co., Ltd.; National Drug Approval No. H10950029; 600 mg) 600 mg orally daily; Calcitriol soft capsules (Catalent Germany Eberbach GmbH; Import Drug Registration Nos. H20140597 & H20140598; 0.25 µg) 0.5 µg orally once daily; and Elcatonin injection (Shandong Luye Pharmaceutical Co., Ltd.; National Drug Approval No. H20040338; 1 mL:10IU)was administered intramuscularly at 1 mL per dose,twice weekly (all injections were administered by trained hospital nurses). Treatment duration: 3 months. Control group: Conventional intervention only.

Observation group: Conventional intervention plus Rǔ-Mò Analgesic Decoction (10 g Frankincense, 10 g Myrrh, 3 g Dragon’s Blood, 6 g Dalbergia wood, 10 g Sappan wood, 10 g Chinese spikenard, 3 g Asarum root, 6 g Ground beetle).Table 2 summarises the key components of Rumo zhentong Yin.

**TABLE 2:** Key Components of Rumo zhentong Yin.

Chinese name	Latin name/English name	Source organism/Plant part	Quantity (g per day)	Components of Rumo zhentong Yin
Ruxiɑng	Olibanum	Boswellia sacra Flück	10	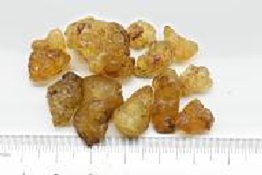
Moyɑo	Myrrha	Commiphora wightii	10	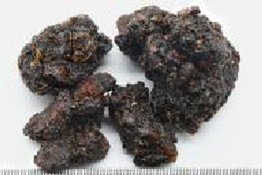
Xuejie	Draconis sanguis	Daemonorops draco Bl	3	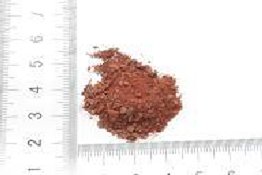
Jiɑngxiɑng	Dalbergiae odoriferae lignum	Dalbergia odorifera T.C.Chen	6	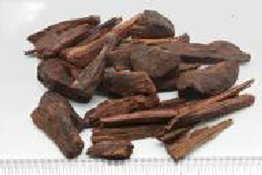
Sumu	Sappan lignum	Biancaea sappan	10	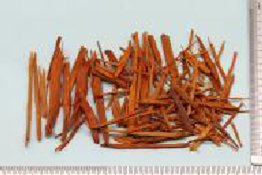
Gɑnsong	Nardostachyos radix et rhizoma	Nardostachys jatamansi (D.Don) DC.	10	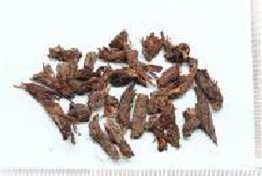
Xixin	Asari radix et rhizoma	Asarum sieboldii miq.	3	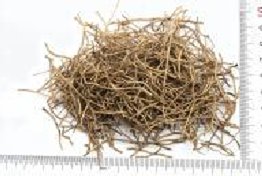
Tubiechong	Eupolyphaga seu polyphaga	Eupolyphaga sinensis	6	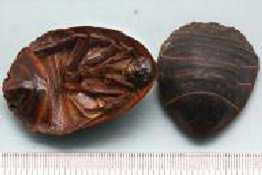
​	​	土鳖虫不是植物,所以这是他的学名不是 source plant	​	​

All herbal ingredients of Rumo Zhentong Yin were provided by the Pharmacy Department of Xi’an Hospital of Traditional Chinese Medicine and met the quality standards of the Chinese Pharmacopoeia (2020 Edition). Each batch underwent hospital incoming quality inspection. Processing was conducted in accordance with hospital standards; impurities and non-medicinal parts were removed. Specifically, only dried roots and rhizomes of Asarum were used, excluding aerial parts.

Prescriptions were decocted centrally in the hospital decoction room with automatic herbal decoction machines under a unified protocol, including standardized soaking, water addition, boiling time and filtration. The finished decoction was sealed and dispensed to participants. Batch inspection was performed to maintain consistent preparation during the trial.

#### Concomitant-therapy rules

Throughout the trial, participants are prohibited from using any concomitant medication or intervention that could interfere with the assessment of efficacy or safety. If a participant has a chronic comorbidity requiring ongoing treatment, all concurrent therapies must be documented in the Case Report Form (CRF).

Throughout the trial, participants are forbidden to use any concomitant medication or intervention that could interfere with the assessment of efficacy or safety. If a participant suffers from a chronic co-morbidity that requires continuous treatment, all concurrent therapies must be documented in the Case Report Form (CRF).

### Outcomes

Primary endpoints: Pain score: Visual Analogue Scale (VAS); Mechanism indicators: 1.Bone-mineral density (BMD); 2.Bone-turnover markers: β-CTX and PINP; 3.Inflammatory cytokines: TNF-α and IL-1.

Secondary endpoints:1.Blood-stasis syndrome score; 2.Hematological indices: serum MCV, RDW-CV, MPV and PDW.

Safety endpoints:Height, blood pressure, 12-lead ECG, routine hematology, liver and renal function tests, and coagulation profile.

### Randomisation and blinding

Simple randomisation was used. The allocation sequence was generated using SPSS version 24.0. Each unique allocation code was placed in a sequentially numbered, opaque, sealed envelope (numbered 1–114) and stored by an independent custodian. After obtaining informed consent, the next envelope in sequence was opened, and the participant was assigned to either the control or treatment group (n = 57 per group). Because the two interventions could not be blinded to participants, only the outcome assessors, data managers, and statisticians were masked to group assignment. In the Case Report Form (CRF), group labels were replaced with the identifiers“A” and “B.” The master randomisation list (containing the sealed code linking A/B to actual groups) was generated by an external statistician using SAS software and is securely stored by the study administrator. It will remain concealed until after final database lock.

### Sample size

This is a parallel, two-arm, superiority RCT. A pilot study (Tong-bi Bushen formula for osteoporosis with kidney-deficiency/blood-stasis pattern) showed mean ± SD VAS improvements of 2.45 ± 0.53 (combination) vs. 2.07 ± 0.53 (Western medicine alone). Using an independent-samples t-test in PASS 15.0.1 (α = 0.05 two-sided, power = 0.95, 1:1 ratio) gave 51 participants per arm; allowing 10% attrition, 114 participants (57 per arm) will be enrolled. Data collection and registration All data will be captured in a paper/e-CRF. Investigators will be trained in patient communication, CRF completion and all rating scales. After consent, baseline demographics, BMD, TCM syndrome score, VAS, bone-turnover markers (β-CTX, PINP), inflammatory cytokines (TNF-α, IL-1), blood-stasis score and haematological indices (MCV, RDW-CV, MPV, PDW) will be collected. Follow-up visits occur every 4 weeks for 3 months. Original data and biological samples will be retained for a minimum of 5 years following the conclusion of the study.

An independent Data Monitoring Committee (DMC) consisting of external clinical epidemiologists, data managers and biostatisticians with no conflicts of interest will review safety, CRF accuracy and participant confidentiality every 6 months and will advise on protocol modification or early termination if warranted.

### Statistical analysis

Statistical analyses will be performed with SPSS 24.0. Continuous variables will be presented as mean ±standard deviation (SD) if normally distributed, or as median (P25, P75) otherwise. Categorical variables will be summarised as frequencies and percentages (*n* [%]). Between-group comparisons of continuous endpoints be conducted using independent-samples t-test (normal) or Mann–Whitney U test (non-normal). Within-group changes over multiple visits will be analysed with repeated-measures ANOVA (normal) or generalised estimating equations (non-normal). If an interaction is significant, simple-effects analysis will follow. Unordered categorical variables will be compared with χ^2^ or Fisher’s exact test. Ordered categorical variables with the rank-sum test. All tests will be two-tailed, with a significance level set at P < 0.05. Missing values will be imputed by last-observation-carried-forward (LOCF) method.

### Trial status

Participant recruitment was initially projected to start in January 2025 and close in June 2026, with follow-up assessments expected to finish in December 2026. However, due to preparatory delays, actual enrollment commenced on 12 September 2025, as registered with the International Traditional Medicine Clinical Trial Registry. Recruitment will continue until the registered closing date of 31 December 2026.

### Ethics and dissemination

The study will be conducted in accordance with the Declaration of Helsinki, the SPIRIT 2013 statement and the SPIRIT-TCM Extension 2018. Ethical a pproval has been obtained from the Medical Ethics Committee of Xi’an Hospital of Traditional Chinese Medicine, affiliated to Shaanxi University of Chinese Medicine (approval No. LLSCYJ2025085). The trial is registered with the International Traditional Medicine Clinical Trial Registry (ITMCTR2026000740). Any protocol amendments will be promptly submitted to the ethics committee for review and approval. Only individuals who provide written informed consent will be enrolled in the study. On completion, the findings will be submitted for publication in a peer-reviewed journal. Clinical insights and trial experience will be shared with participants and the wider public through conferences and publications. Authorship of the final report will be determined by individual contributions to the research.

## Discussion

The latest national epidemiological survey shows that the prevalence of osteoporosis among Chinese residents aged ≥50 years is 19.2% (32.1% in women, 6.9% in men), rising to 32% in those ≥65 years (51.6% in women, 10.7% in men). These figures correspond to roughly 90 million affected individuals nationwide and an estimated annual direct medical cost of 174.5 billion RMB, making osteoporosis a major public-health challenge (National Center for Chronic Noncommunicable Diseases, 2021; Si et al., 2018). Osteoporotic pain (OPP) is the most common clinical symptom prompting medical consultation. Apart from chronic low-back pain, OPP is frequently accompanied by sensory/motor dysfunction, sleep disturbance and psychological distress (Si et al., 2018; Nees and Becker, 2017). As bone mass declines and micro-architecture deteriorates, intermittent pain may become continuous, markedly impairing quality of life.

Current Western approaches to managing osteoporotic pain include anti-osteoporosis medications, NSAIDs, opioids and non-pharmacological measures such as exercise therapy, nerve block, radio-frequency ablation, percutaneous vertebroplasty or kyphoplasty (Shima et al., 2016; Nees and Becker, 2017; Gongyi, 2003; Valet et al., 2009; Vellucci et al., 2018). However, none of these options is fully satisfactory. Anti-osteoporosis agents can provide partial pain relief but are associated with adverse effects including fever, oedema, gastrointestinal ulceration, osteonecrosis of the jaw or atypical femoral fracture (Gong et al., 2003). NSAIDs increase the risk of gastrointestinal bleeding and cardiovascular events and interfere with fracture healing (Cummings et al., 1998). Long-term opioid use is linked to an elevated risk of falls, fractures, endocrine dysfunction and mood deterioration (Lisowska et al., 2018; Liberman et al., 1995). Minimally invasive procedures are safe but expensive and highly selective. Given these limitations, an effective, safe and affordable treatment is urgently needed.

In recent years, the therapeutic advantages of Traditional Chinese Medicine (TCM) in the management of osteoporotic pain (OPP) have become increasingly evident. By delaying bone loss, regulating bone metabolism, alleviating pain symptoms, and simultaneously addressing associated psychological and somatic discomforts, TCM offers a more holistic and patient-centered approach to OPP treatment.Wu Weiping et al. (Wu and Chao, 2009) enrolled 55 patients with OPP and administered a TCM regimen aimed at tonifying the kidney, strengthening the spleen, promoting blood circulation, and removing blood stasis, in addition to conventional therapy. The overall response rate in the treatment group was significantly higher than that in the control group. Similarly, Wang Wenzheng et al. (Wang et al., 2016) combined Shufu Tablets (Shufu Pian), which are known for their blood-activating and stasis-resolving properties, with Bushen Zhuanggu Tang (Kidney-Tonifying and Bone-Strengthening Decoction). Their findings demonstrated that this combination therapy produced superior improvements in nocturnal pain, daytime resting pain, and activity-related pain (e.g., pain during turning over) in patients with osteoporosis.

From a TCM theoretical perspective, the principal pathological mechanisms underlying OPP involve “pain due to obstruction” (Bu Tong Ze Tong) and “pain due to lack of nourishment” (Bu Rong Ze Tong). These mechanisms arise from liver–kidney deficiency, depletion of marrow and essence, qi stagnation and blood stasis, as well as the invasion of external pathogenic factors. Among these, blood stasis serves not only as a key etiological factor but also as a pathological consequence in the progression of osteoporotic pain.

“Rǔ-Mò Zhèn-Tòng-Yǐn” (Frankincense-Myrrh Analgesic Decoction) is an empirical formula created by the national TCM master Yao Shu-jin for blood-stasis pain. Its bio-active components exhibit potent anti-inflammatory and analgesic properties through multiple molecular pathways.

Frankincense triterpenoid (3-oxo-tirucosta-8,24-dien-21-oic acid, KTDA) and myrrh sesquiterpenoid (2-methoxy-5-acetyl-furanogermacr-1 (10)-en-6-one, FSA) suppress MAPK/AKT phosphorylation, inhibit NF-κB nuclear translocation and downregulate IL-1β and IL-6 mRNA (Lihui et al., 2022).Acetyl-11-keto-β-boswellic acid (AKBA) reduces Ti-particle-induced osteolysis and promotes osteoblast differentiation via GSK-3β/β-catenin (Wang, 2020). Asarum alcohol extract decreases NO, PGE_2_ and MDA, elevates SOD and activates GABA_a_ receptors to produce analgesia (Yuan and Sun, 2009; Liu et al., 2021). Ground beetle (Eupolyphaga) suppresses TRPV1 expression, mitigates neuropathic pain, increases osteoblast number and accelerates callus mineralisation (Liguo et al., 2015). Dragon’s-blood chalcones inhibit osteoclast formation via p38/MAPK, JNK and Keap1/Nrf2/ARE pathways (Liu, 2019).

Collectively, these pharmacological actions support the potential of Rǔ-Mò Zhèn-Tòng-Yǐn to alleviate osteoporotic pain OPP. However, high-quality, well-designed randomised clinical trials evaluating TCM formulas as add-on therapy are scarce.

## Limitations of the present trial

The study does not include dose-escalation or de-escalation arm—precluding exploration of dose–response relationships. Furthermore, all participating sites are located in Xi’an, limiting generalizability of the results. Larger, multi-centre studies with diverse populations are warranted. This prospective, randomised, single-blind, controlled trial will evaluate the efficacy and safety of Rǔ-Mò Zhèn-Tòng-Yǐn combined with standard Western therapy versus standard therapy alone in patients with OPP. The primary end-points are improvement in BMD and pain relief. Secondary end-points include changes in bone-turnover markers, inflammatory cytokines and TCM syndrome scores. The findings are expected to provide robust evidence for the integrative management of osteoporotic pain.

## Data Availability

The raw data supporting the conclusions of this article will be made available by the authors, without undue reservation.
